# Expression of Bruton’s Tyrosine Kinase Reflects Immune Cells Infiltration and Cell Proliferation in Breast Cancer

**DOI:** 10.14740/wjon2708

**Published:** 2026-03-05

**Authors:** Tamrah AlRammah, Rongrong Wu, Kohei Chida, Kei Kawashima, Kenichi Hakamada, Takashi Ishikawa, John M.L. Ebos, Kazuaki Takabe

**Affiliations:** a Department of Surgical Oncology, Roswell Park Comprehensive Cancer Center, Buffalo, NY 14263, USA; b Department of General Surgery, Diriyah Hospital, Riyadh Third Cluster, Ministry of Health, Riyadh, Saudi Arabia; c Department of Breast Surgery and Oncology, Tokyo Medical University, Tokyo 160-8402, Japan; d Department of Gastroenterological Surgery, Hirosaki University Graduate School of Medicine, Hirosaki 036-8562, Japan; e Department of Gastroenterological Surgery, Yokohama City University Graduate School of Medicine, Kanagawa 236-0004, Japan; f Department of Cancer Genetics and Genomics, Roswell Park Comprehensive Cancer Center, Buffalo, NY, USA; g Department of Immunology, Roswell Park Comprehensive Cancer Center, Buffalo, NY 14263, USA; h Department of Surgery, University at Buffalo Jacobs School of Medicine and Biomedical Sciences the State University of New York, Buffalo, NY 14263, USA; i Division of Digestive and General Surgery, Niigata University Graduate School of Medical and Dental Sciences, Niigata 951-8520, Japan; j Department of Breast Surgery, Fukushima Medical University School of Medicine, Fukushima 960-1295, Japan; k These authors contributed equally to this manuscript.

**Keywords:** Breast cancer, BTK, Immunology, Molecular biology, Neoadjuvant chemotherapy, Survival

## Abstract

**Background:**

Bruton’s tyrosine kinase (BTK) is a downstream mediator in B-cell receptor (BCR) signaling and is essential for B-cell differentiation and proliferation. BTK inhibitors are approved and in clinical use for hematological cancers such as lymphoma and leukemia, with testing underway in solid tumors. Because BTK is expressed in myeloid-derived suppressor cells (MDSCs) known to worsen breast cancer (BC) outcomes, we investigated the clinical relevance of BTK expression in multiple BC subtypes as a predictor of progression and/or response to treatment.

**Methods:**

We performed an integrative transcriptomic analysis of tumor BTK expression across three large BC cohorts (The Cancer Genome Atlas (TCGA), Molecular Taxonomy of Breast Cancer International Consortium (METABRIC), and Sweden Cancerome Analysis Network-Breast (SCAN-B); total n = 5,240), 10 neoadjuvant chemotherapy (NAC) datasets, and the I-SPY2 neoadjuvant immunotherapy trial cohort. Gene set enrichment analysis (GSEA) and xCell deconvolution were used to evaluate associations with cell proliferation, immune infiltration, and tumor microenvironment (TME) composition while single-cell sequencing datasets (SCP1039, SCP1106) were used to identify BTK-expressing cell populations. Survival analyses were performed using Kaplan-Meier and log-rank tests.

**Results:**

BTK levels were the highest in triple-negative BC (TNBC) among the subtypes and unexpectedly drove stronger proliferation gene set enrichment in estrogen receptor-positive (ER^+^)/human epidermal growth factor receptor 2-negative (HER2^–^) tumors (e.g., MITOTIC_SPINDLE, G2M_CHECKPOINT). However, BTK expression did not correlate with American Joint Committee on Cancer (AJCC) stage or overall, disease-free, or disease-specific survival across cohorts or molecular subtypes. Notably, BTK-high tumors showed robust enrichment of immune pathways (interferon gamma (IFN-γ) response, interleukin-6 (IL-6)/Janus kinase (JAK)/signal transducer and activator of transcription 3 (STAT3), tumor necrosis factor-α (TNF-α) signaling) and exhibited elevated tumor-infiltrating leukocyte and lymphocyte fractions, increased cytolytic activity, and greater abundance of myeloid and lymphoid cell populations. BTK expression was not consistently associated with NAC response as only one of 10 datasets (GSE25066) showed a weak association within ER^+^ and HER2-positive subtypes. Similarly, BTK levels did not predict response in I-SPY2 patients receiving durvalumab plus olaparib, despite strong correlations with programmed cell death protein 1/programmed cell death ligand 1 (PD-1/PD-L1) expression. Single-cell analysis localized BTK transcripts primarily to myeloid and B cells.

**Conclusion:**

BTK expression in BC reflects a proliferative and immune-active TME, particularly in TNBC and HER2-positive subtypes, but lacks prognostic or predictive value for NAC or PD-L1-based immunotherapy response.

## Introduction

Bruton’s tyrosine kinase (BTK) is involved in B-cell maturation and survival through its critical role in B-cell receptor (BCR) signaling and is an established therapeutic target for the treatment of blood cancers. BTK is a driver of B cell-derived malignancies such as chronic lymphocytic leukemia (CLL) and mantle cell lymphoma, where the standard therapies now include BTK small molecule inhibitors ibrutinib, acalabrutinib, and zanubrutinib [1, 2]. Mechanistically, BTK is involved in multiple signaling programs in cells. BTK can transduce signals from Toll-like receptors (TLRs) through myeloid differentiation primary response 88 (MYD88) and interferon regulatory factor 3 (IRF3), and via chemokine receptors where Gα/βγ subunits can directly engage BTK (PH/TH domains) and/or activate phosphatidylinositol 3-kinase (PI3K) upstream. Active BTK can phosphorylate PLCγ2 to produce IP3 (Ca^2+^ flux/NFAT) and DAG and can activate PI3-AKT-mTORC1 which, in turn, supports B-cell activation, proliferation, differentiation, and survival [3–6]. Together, BTK converges on nuclear factor-κB (NF-κB), mitogen-activated protein kinase (MAPK), and protein kinase B (AKT) pathways to drive antibody secretion, class-switch recombination, and pro-inflammatory cytokine production [7].

BTK is now known to function well beyond B lymphocytes and play a role in a wide range of innate immune cells that include macrophages, neutrophils, mast cells, dendritic cells (DCs), and myeloid-derived suppressor cells (MDSCs) [8]. Through these signaling pathways, BTK promotes cytokine release, inflammasome activation, and antigen presentation, thereby influencing both pro-inflammatory and immunosuppressive mechanisms [9].

Interestingly, ibrutinib, a BTK inhibitor, was shown to prevent drug resistance and promote BC cell death *in vitro* and *in vivo* studies [10, 11]. BTK blockade was found to suppress erythroblastic leukemia viral oncogene homolog B (ERBB) signaling including epidermal growth factor receptor (EGFR), human epidermal growth factor receptor 2 (HER2), human epidermal growth factor receptor 3 (HER3), and human epidermal growth factor receptor 4 (HER4), which, in turn, rendered HER2^+^ tumors more sensitive to ibrutinib than luminal and triple-negative tumors in preclinical setting [10, 11].

In this study, we studied the clinical relevance of BTK expression in BC patients using large independent BC cohorts that link transcriptome and clinical data: The Cancer Genome Atlas (TCGA), Molecular Taxonomy of Breast Cancer International Consortium (METABRIC), Sweden Cancerome Analysis Network-Breast (SCAN-B), and I-SPY2 neoadjuvant immunotherapy cohorts. Leveraging our established expertise in integrative *in silico* translational research [12–18], we applied a comprehensive analytical approach to evaluate the biological and clinical significance of BTK expression. We hypothesized that BTK levels are associated with cancer cell proliferation, immunosuppression, treatment resistance, and worse survival outcomes.

## Materials and Methods

### Ethical compliance

Institutional review board (IRB) approval at Roswell Park Comprehensive Cancer Center (Buffalo, New York, USA) was waived as publicly available deidentified databases were used.

### Data retrieval and processing

All data were obtained from cBioportal [19], Gene Expression Omnibus (GEO) database [20], or Single Cell Portal (SCP) [21]. All GEO datasets were downloaded using the R package GEOquery (version 2.78). For microarray-based datasets, expression values were mapped to HUGO gene symbols using the corresponding platform annotation files. When multiple probes were annotated to the same gene, the gene-level expression value was calculated as the mean of all probes assigned to that gene. For GSE123845 and the I-SPY2 cohort (GSE173839), gene-level normalized expression data were provided and were used directly without additional normalization or probe-level summarization. Breast cancer (BC) staging followed the guidelines set by the American Joint Committee on Cancer (AJCC).

The main BC cohorts that we examined were TCGA BC cohort (n = 936): estrogen receptor-positive (ER^+^)/human epidermal growth factor receptor 2-negative (HER2^–^) (n = 592, 63%), HER2-positive (n = 184, 20%), triple-negative BC (TNBC, n = 160, 18%) [22], the METABRIC cohort (n = 1,094): ER^+^/HER2^−^ (n = 1,355, 71%), HER2-positive (n = 236, 12%), TNBC (n = 313, 17%) [23], and the SCAN-B (GSE96058) cohort (n = 2,824): ER^+^/HER2^−^ (n = 2277, 81%), HER2-positive (n = 392, 14%), TNBC (n = 155, 5%) [24]. For the TCGA-BRCA cohort, we manually associated the biomarker expression status and subtyping from the pathology reports in the Text Information Extraction System (TIES) Cancer Research Network, which is a federated network that facilitates data and biospecimen sharing among member institutions [20], including TCGA, in cases where this information was missing in cBioPortal, ensuring accurate identification and sufficient sample sizes.

Other BC cohorts examined in this research included those involving patients treated with immunotherapy (I-SPY2; GEO dataset GSE173839 (n = 105) [25]) or neoadjuvant chemotherapy (NAC): GSE16446 (n = 120) [26], GSE20194 (n = 278) [27], GSE20271 (n = 178) [28], GSE22226 (n = 130) [29], GSE22358 (n = 153–158) [30], GSE25066 (n = 508) [26], GSE32603 (n = 248 ) [31], GSE50948 (n = 156) [32], GSE123845 (n = 227) [33], and GSE163882 (n = 222) [34]. For single-cell (sc) RNA data, we used SCP datasets SCP1039 [35] (26 primary tumors, including 11 ER^+^, five HER2-positive, and 10 TNBC tumors, total 130,246 cells) and SCP1106 (five TNBCs, total 24,271 cells) [36].

### Molecular and cellular features of tumors

For TCGA cohort, scores for cell proliferation, homologous recombination defects, mutation rates, neoantigen presence, and immune activity were obtained from the study of Thorsson et al [37]. For all cohorts, the composition of infiltrating immune and stromal cells in the tumor microenvironment (TME) were estimated from gene expression data using xCell algorithm [38].

### Gene set enrichment analysis (GSEA)

GSEA was employed to evaluate differences in pathway level gene expression profiles between study groups. Pathway enrichment analysis was conducted using the 50 Hallmark gene sets from the Molecular Signatures Database (MSigDB), applying the standard pathways developed by the Broad Institute [39]. Pathway significance was assessed using the normalized enrichment scores (NES), and multiple testing correction was performed using the false discovery rate (FDR) method, accounting for both gene size and multiple comparisons. Gene sets with an FDR below 0.05 were considered significantly enriched.

### Estimation of MDSCs infiltration in the bulk tumor

MDSCs abundance was inferred from bulk tumor transcriptomic data using multiple previously published gene-expression signatures. These signatures were originally reported by Wang et al (pan-cancer MDSC signature [11]), Alshetaiwi et al (BC-specific MDSC signature [40]), Cristecu et al (monocytic MDSC (mMDSC) and granulocytic MDSC (gMDSC) signatures [41]), and Kobayashi et al (PMN-MDSC signature [42]). For each cohort, gene set variation analysis (GSVA) was performed to estimate relative enrichment of each MDSC-associated gene signature across samples, using the fgsea package in R.

### Spatial transcriptomics analysis

Publicly available Visium HD Spatial Gene Expression data of a human invasive ductal carcinoma (fixed-frozen) was downloaded from the 10x Genomics website [43]. The dataset includes spatial gene expression profiles and matched hematoxylin and eosin (H&E)-stained histology images. Gene expression data were analyzed at the Visium HD binned resolution, with each spatial bin measuring approximately 8 µm in diameter. Expression matrices were log-normalized prior to analysis. Spatial bins were annotated to major cell types based on dominant expression of cell type–specific marker genes. *BTK* expression was visualized on tissue sections after scaling and upper-quantile truncation.

### Statistical analysis

To investigate the differences in the clinicopathological and biological features between low and high BTK expression, patients were divided into low and high BTK groups by the median value within each cohort. The median values for each BC subtype were used, resulting in different median values for each subtype and creating separate low and high groups within each subtype. Although median-based dichotomization is arbitrary and may raise concerns to introduce heterogeneity and/or reduce analytical rigor, there is no biologically or clinically validated threshold to define high versus low expression in BTK, and many of the previously published studies investigating the clinical significance of specific gene expression levels have commonly employed median-based stratification [15, 44–46].

Statistical analyses were conducted using R (version 4.5.1) with these packages: ggplot2 (3.5.2), ggpubr (0.6.1), ggrepel (0.9.6), ggsignif (0.6.4), gtsummary (2.3.0), Matrix (1.7-3), Seurat (version 5.3.0), SeuratObject (5.1.0), survival (3.8-3), survminer (0.5.0), and patchwork (1.3.1). To investigate the differences in the clinicopathological and biological features between low and high BTK expression, patients were divided into low and high BTK groups by the median value within each cohort. The median values for each BC subtype were used, resulting in different median values for each subtype and creating separate low and high groups within each subtype. Comparisons between the two groups were conducted using the Kruskal-Wallis and Wilcoxon signed-rank tests. For survival analysis, the Kaplan-Meier method with log-rank test was used. A P-value threshold of 0.05 was used to detect statistical significance.

## Results

### BTK expression was not associated with cancer stage nor survival in BC

**Figure 1:**
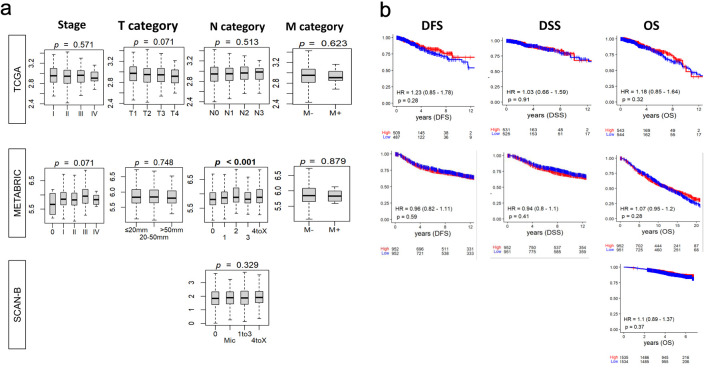
Relationship between tumor BTK expression and breast cancer aggressiveness and survival. BTK mRNA expression in three cohorts was examined. (a) Tukey boxplots of BTK expression among tumors categorized by overall AJCC stage and status for tumor size (T), lymph node (N), and metastasis (M). P values from Kruskal-Wallis test are shown. (b) Estimated survival (Kaplan-Meier) with log-rank test P value and hazard ratio (HR) of disease-free survival (DFS), disease-specific survival (DSS), and overall survival (OS) for groups of patients with low and high BTK expression within the cohorts, using median value as cut-off.

Given the clinical relevance of BTK expression in leukemia, it was of interest to investigate the relationship between BTK expression and BC aggressiveness. To this end, we measured the BTK mRNA expression by the AJCC staging in BC cohorts. There was no significant difference in BTK expression by any of the categories (tumor, nodal, metastatic) in AJCC staging in TCGA, METABRIC or SCAN-B cohorts, except for N category in METABRIC (P = 0.001) (Fig. 1a). To evaluate the prognostic significance of BTK expression in BC, we analyzed its association with survival (Fig. 1b). There was no significant difference in disease-free survival (DFS), disease-specific survival (DSS), nor overall survival (OS) across three large independent cohorts, TCGA, METABRIC, and SCAN-B, by the BTK expression. Further, we analyzed BTK expression and survival by subtype (ER^+^HER2^–^/HER2-positive/TNBC), with no significant association with survival outcome observed (Supplementary Material 1, wjon.elmerpub.com).

### BTK level was high in TNBC, whereas it was associated with cell proliferation in ER^+^HER2^–^ and HER2-positive subtypes

**Figure 2:**
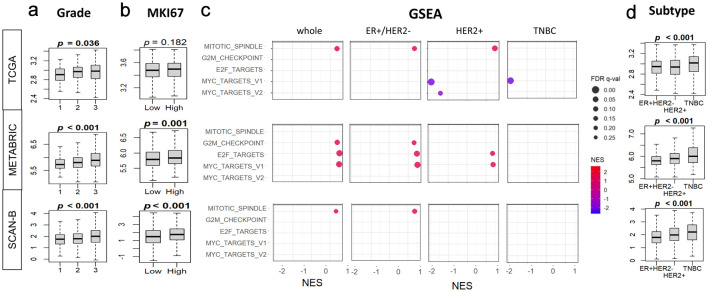
Associations of tumor BTK expression with Nottingham histological grade, MKI67 gene expression, and cell proliferation pathways in breast cancer. BTK mRNA expression in three cohorts was examined. (a) Tukey boxplots of BTK expression in different histological grades of breast cancer. (b) Tukey boxplots of tumor MKI67 expression in tumor groups with low and high BTK expression, using median value as cut-off. (c) Gene set enrichment analysis (GSEA) of cell proliferation-related Hallmark gene sets (E2F targets, G2M checkpoint, Myc targets v1 and v2, mitotic spindle), comparing tumor groups with low and high BTK expression, using median value as cut-off. (d) Tukey boxplots of BTK expression in different cancer subtypes. (e) GSEA analyses for different cancer subtypes as in (c). GSEA NES and FDR values were calculated using GSEA software. P values shown with boxplots are from Kruskal Wallis or Mann-Whitney U tests. NES: normalized enrichment score; FDR: false discovery rate.

Since BTK expression was not associated with BC survival, we next asked whether it is associated with BC cell proliferation. We observed that BTK levels significantly correlated with Nottingham histological grade consistently across all the three cohorts, TCGA (P = 0.036), METABRIC (P = 0.021), and SCAN-B (P = 0.001) (Fig. 2a). The proliferation marker MKI67 was significantly elevated in high-BTK BC in the METABRIC and SCAN-B cohorts (both P = 0.001), but not in TCGA (P = 0.182) (Fig. 2b). Then we looked at the proliferation-related Hallmark gene sets in the overall cohort, comparing high- and low-BTK tumors by GSEA, with FDR < 0.25 indicating significant enrichment. Significant enrichment to high-BTK BC was observed for mitotic spindle in TCGA (NES = 1.36) and SCAN-B (NES = 1.40), and for G2M checkpoint, E2F targets, and MYC targets v1 in METABRIC (NES = 1.39, 1.50, and 1.49, respectively (Fig. 2c) in whole BC including all subtypes. Interestingly, GSEA of cell proliferation-related gene sets were quite different by the subtypes. ER^+^HER2^–^ subtype enriched the same cell proliferation-related gene sets as the whole BC, mitotic spindle in TCGA (NES = 1.43) and SCAN-B (NES = 1.35), and in G2M checkpoint (NES = 1.32), E2F targets (NES = 1.42), and MYC targets v1 (NES = 1.47) in METABRIC. In the HER2-positive subtype, high-BTK BC enriched only mitotic spindle in TCGA (NES = 1.45) and E2F targets and MYC targets v1 in METABRIC (NES = 1.36 and 1.39, respectively), and MYC targets v1 and v2 were enriched to low-BTK BC in TCGA (NES = −1.83 and −1.33, respectively). GSEA was remarkably different in TNBC, where none of the cell proliferation-related gene sets enriched to high-BTK BC, and MYC targets v1 enriched to low-BTK BC in TCGA (NES = −1.85) (Fig. 2d). Across TCGA, METABRIC, and SCAN-B cohorts, BTK mRNA expression was significantly higher in TNBC compared with ER^+^HER2^–^ and HER2-positive tumors (all P < 0.001) (Fig. 2d).

### High BTK expression was associated with activation of immune pathways but not with mutations and neoantigens

**Figure 3:**
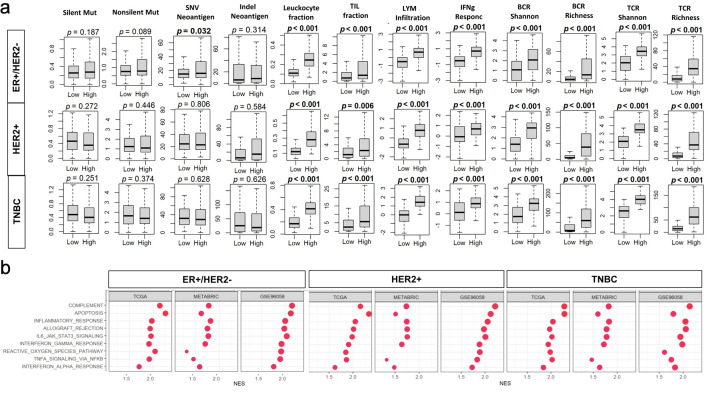
Associations of tumor BTK expression with mutation burden and immune cell infiltration in breast cancer. Tumor groups with low and high BTK expression, using median value as cut-off, were compared. (a) Tukey boxplots of mutation- and immune infiltration-related features in the TCGA cohort. BCR: B-cell receptor; LYM: lymphocyte; Mut: mutation; SNV: single nucleotide variant; TCR: T-cell receptor; TIL: tumor-infiltrating lymphocyte. (b) Gene set enrichment analysis (GSEA) comparing high- and low-BTK groups for three cohorts across whole cohorts or by subtypes. Immune-related Hallmark gene sets are examined with GSEA software. NES: normalized enrichment score; FDR: false discovery rate.

Given that the relationship between BTK and cell proliferation differed significantly by the subtypes, it was of interest to see the relationship between BTK and mutation burden of BC by the subtype. BTK expression was not significantly associated with the burden of silent mutations, non-silent mutations, single nucleotide variant (SNV) or indels neoantigens regardless of BC subtype except for SNV neoantigens for ER^+^HER2^–^ subtype (P = 0.032). Across all three subtypes, BTK-high tumors demonstrated significantly higher leukocyte fraction, tumor-infiltrating lymphocyte (TIL) fraction, lymphocyte infiltration score, interferon gamma (IFN-γ) response, BCR diversity (Shannon index and richness), and T-cell receptor (TCR) diversity (Shannon index and richness) compared with BTK-low tumors (all P < 0.001, except for leukocyte fraction in HER2^+^ (P = 0.006)). These differences were mostly pronounced in TNBC (Fig. 3a). To further define activated pathways, we assessed Hallmark immune-related gene sets in the TCGA, METABRIC, and SCAN-B cohorts among all BC subtypes. BTK-high tumors exhibited robust enrichment of immune and inflammatory pathways in the overall population and within each BC subtype. Consistently enriched pathways included allograft rejection, apoptosis, complement, interleukin-6 (IL-6)/Janus kinase (JAK)/signal transducer and activator of transcription 3 (STAT3) signaling, inflammatory response, IFN-γ response, reactive oxygen species pathway, and tumor necrosis factor-α (TNF-α) signaling via NF-κB (NES = 1.5–2.2 and FDR < 0.05 for all). The strongest enrichments were observed for allograft rejection and IFN-γ response (NES = 2.0–2.2), particularly in TNBC and HER2-positive tumors (Fig. 3b).

**Figure 4:**
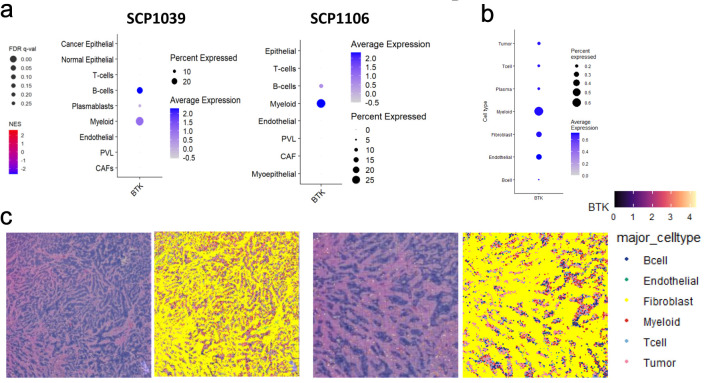
Single cell sequencing and spatial transcriptomic analyses of BTK expressing cells in breast cancer. (a) Heatmaps and dot plots represent BTK expression patterns across various cell types in SCP1039 and SCP1106 single cell sequencing cohorts. PVL: perivascular-like cells; CAF: cancer-associated fibroblast. The dot size corresponds to the percentage of cells expressing BTK, while the color intensity represents the average expression level of BTK within those cell populations. (b) Heatmaps and dot plots displaying the expression of BTK gene in various cell populations, including tumor cells, T cells, plasma cells, myeloid, fibroblast, endothelial and B cells. The dot size corresponds to the percentage of cells expressing BTK, while the color intensity represents the average expression level of BTK within those cell populations. (c) Spatial mapping of BTK gene expression and major cell types in human breast cancer tissue. The image displays data from Visium HD gene expression library generated from a human breast tissue sample (fixed frozen) with invasive ductal carcinoma, sourced from a 10x Genomics webpage. The left panel shows the expression levels of the BTK gene, color-coded from low (purple/blue) to high (yellow/red). The right panel shows the spatial distribution of annotated major cell types, including B cells, endothelial cells, fibroblasts, myeloid cells, T cells, and tumor cells. Data were binned to an 8 × 8 µm resolution for visualization and analysis.

To clarify which cells are responsible for BTK expression in the bulk tumor, we examined the BTK expression in two independent single cell sequence BC cohorts, SCP1039 and SCP1106. BTK transcripts were detected in B and myeloid cells, with approximately 15% of B cells and 30% of myeloid cells expressing BTK, while other cell types showed negligible expression (Fig. 4a). To delineate the cellular source and spatial context of BTK expression within the TME, BTK expression was analyzed across major cell population using Visium HD spatial transcriptomic data. Among major cellular compartments, myeloid cells exhibited the highest BTK expression. Moderate BTK expression was also observed in fibroblasts and endothelial cells, whereas tumor cells, T cells, B cells, and plasma cells showed minimal expression (Fig. 4b). Spatial mapping of annotated cell types onto matched histology section demonstrated that myeloid cells were preferentially localized around tumor cell region, often forming peritumoral niches near malignant cells (Fig. 4c). This analysis confirmed that the BTK signals arise predominantly from peritumoral myeloid and B cells clusters, rather than tumor intrinsic expression. This spatial enrichment, together with cell type expression pattern, indicates that BTK is predominantly associated with peritumoral myeloid populations within the TME.

### High BTK expression was associated with a myeloid- and lymphocyte-enriched immune microenvironment and elevated cytolytic activity

**Figure 5:**
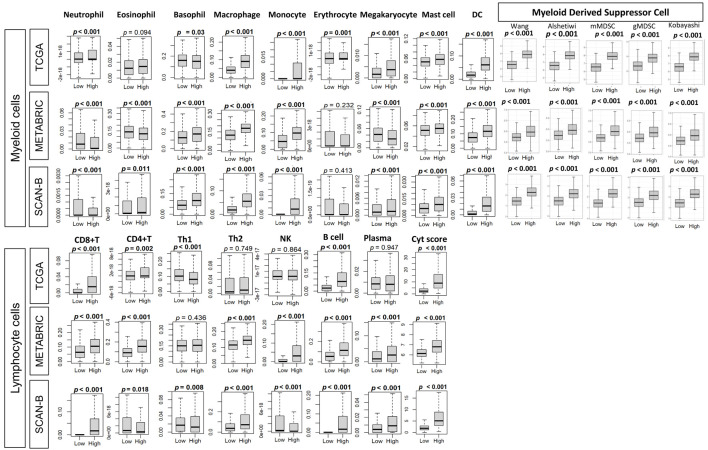
Association of tumor BTK expression with immune cell composition and with MDSC gene expression signatures. Immune cell composition was estimated from tumor transcriptomes with xCell algorithm and compared between tumor groups with low and high BTK expression, using median value cut-off, for three cohorts. Tukey boxplots are shown for various immune cell-types and MDSC gene expression signatures, including Wang, Alshetaiwi, Cristecu granulocytic MDSC (gMDSC), Cristecu monocytic MDSC (mMDSC), and Kobayashi. Mann-Whitney U test was used to determine P values. MDSCs: myeloid derived suppressor cells; Cyt: cytolytic activity score; DC: dendritic cell; NK: natural killer cell; Th: T helper cell.

Given this association between BTK expression and immune activity in TME, we further analyzed the types of immune cell infiltrations in TME using the xCell algorithm. Among myeloid cell subsets, neutrophils, macrophages, monocytes, mast cells, and DCs were significantly more abundant in BTK-high tumors across all three datasets (all P < 0.001). Basophil levels were also elevated in BTK-high tumors in all TCGA, METABRIC, and SCAN-B cohorts (P ≤ 0.03), whereas eosinophil infiltration was significantly higher in METABRIC (P < 0.001) and SCAN-B (P = 0.011) but not in TCGA (P = 0.094). Erythrocytes were enriched only in TCGA (P = 0.001), while megakaryocyte abundance was consistently elevated across cohorts (all P < 0.001) (Fig. 5). Within the lymphocyte compartment, CD8^+^ T-cell levels were markedly higher in BTK-high tumors in all datasets (all P < 0.001), accompanied by increased CD4^+^ T-cell infiltration in TCGA (P = 0.002), METABRIC (P < 0.001), and SCAN-B (P = 0.018). Th1 cells were elevated in TCGA (P < 0.001) and SCAN-B (P = 0.008), but not in METABRIC (P = 0.436), whereas Th2 cells were significantly higher in METABRIC (P < 0.001) and SCAN-B (P < 0.001), but not in TCGA (P = 0.749). NK cell infiltration was increased in METABRIC and SCAN-B (both P < 0.001), but unchanged in TCGA (P = 0.864). B-cell abundance was consistently higher in BTK-high tumors across all cohorts (all P < 0.001), and plasma cell infiltration was elevated in METABRIC and SCAN-B (both P < 0.001) but not in TCGA (P = 0.947). The cytolytic score was consistently higher in BTK-high tumors across all cohorts (P < 0.001), implicating enhanced immune-cell killing. These findings demonstrate that BTK-high BCs exhibit a consistent enrichment of both myeloid- and lymphocyte-lineage immune cells, coupled with elevated cytolytic activity, indicative of an immune-active TME.

### Infiltration of MDSCs correlated with BTK expression consistently in all the subtypes across all three cohorts

Across three independent BC cohorts (TCGA, METABRIC, and SCAN-B), we observed consistent and significant infiltration of MDSC in the BTK high group compared with BTK low group, irrespective of the score used, which were Wang et al (pan-cancer MDSC signature [11]), Alshetaiwi et al (BC-specific MDSC signature [40]), Cristecu et al (mMDSC and gMDSC signatures [41]), and Kobayashi et al (PMN-MDSC signature [42]) (all P < 0.001, Fig. 5). Further, correlation of BTK expression and MDSC infiltration was consistent across all BC subtypes (ER^+^/HER2^–^, HER2^+^, and TNBC, Supplementary Material 2, wjon.elmerpub.com). The fact that elevated MDSC scores were closely aligned with high BTK expression suggests a mechanistic link between BTK signaling and the establishment of an immune suppressor microenvironment.

### BTK-high tumors were not associated with response to NAC

**Figure 6:**
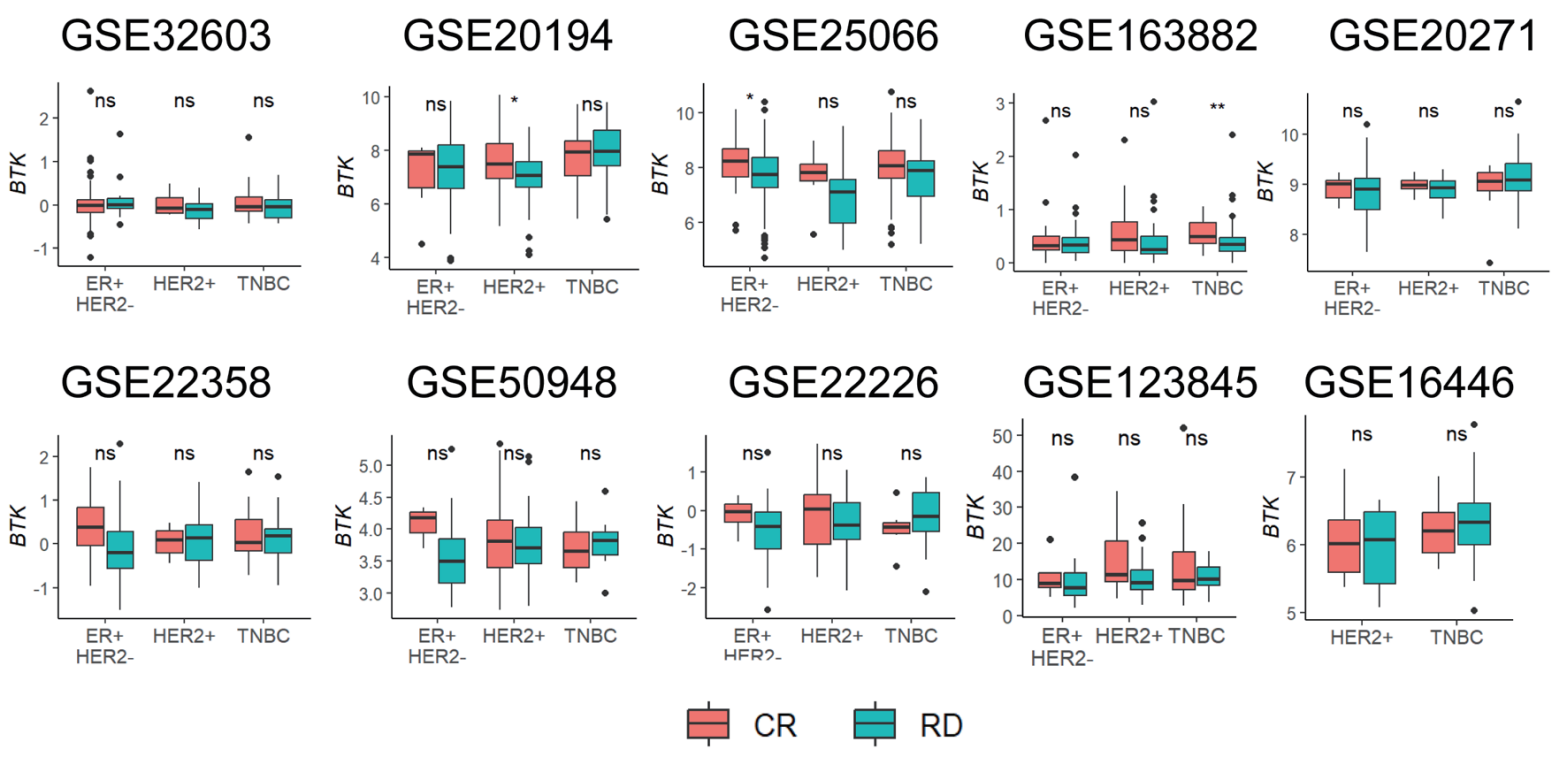
Association of pre-treatment tumor BTK expression with response to neoadjuvant chemotherapy. Tukey boxplots of BTK expression are shown for patient groups with either complete response (CR) or residual disease (RD) after neoadjuvant chemotherapy for different cancer subtypes. Mann-Whitney U test: *P < 0.05, **P < 0.005, ns > 0.05.

Given that BTK expression correlated with cell proliferation, infiltration of the immune cells and enhanced immune cell killing, we expected BTK expression to be associated with response to NAC. This analysis included 10 independent BC cohorts with available gene expression profiles and treatment response data, including NAC regimens that varied across the studies. The regimens were: for GSE16446: epirubicin; for GSE32603: doxorubicin, cyclophosphamide, and paclitaxel (AC-T); for GSE20194, GSE20271 and GSE25066: 5-fluorouracil and AC-T regimens; for GSE22226, GSE123845, and GSE163882: anthracyclines and taxanes, with or without trastuzumab; for GSE22358: capecitabine and docetaxel, with or without trastuzumab; for GSE50948: AC-T regimen, methotrexate, and 5-fluorouracil, with or without trastuzumab. Collectively, these data sets encompassed all three major molecular subtypes of BC. Across these 10 datasets, GSE25066 showed that BTK expression was significantly associated with achieving pathological complete response (pCR) in the HER2-positive (P = 0.014), but not in ER^+^ (P = 0.050) or TNBC (P = 0.69) subtype. No significant associations between BTK expression and pCR were observed in the remaining nine cohorts (Fig. 6). Unexpectedly, these results indicate that although BTK-high tumors exhibit strong immune-related signatures, tumors that achieving pCR did not show statistically higher BTK levels. The only observed benefit was restricted to a single cohort and specific subtypes, suggesting that BTK-related immune activation does not necessarily translate into enhanced chemotherapy responsiveness.

### High BTK cell infiltration was associated with myeloid enriched microenvironment, immune gene expression signature, immune checkpoint molecule expressions but was not related to response to immunotherapy in I-SPY2 trial patients

**Figure 7:**
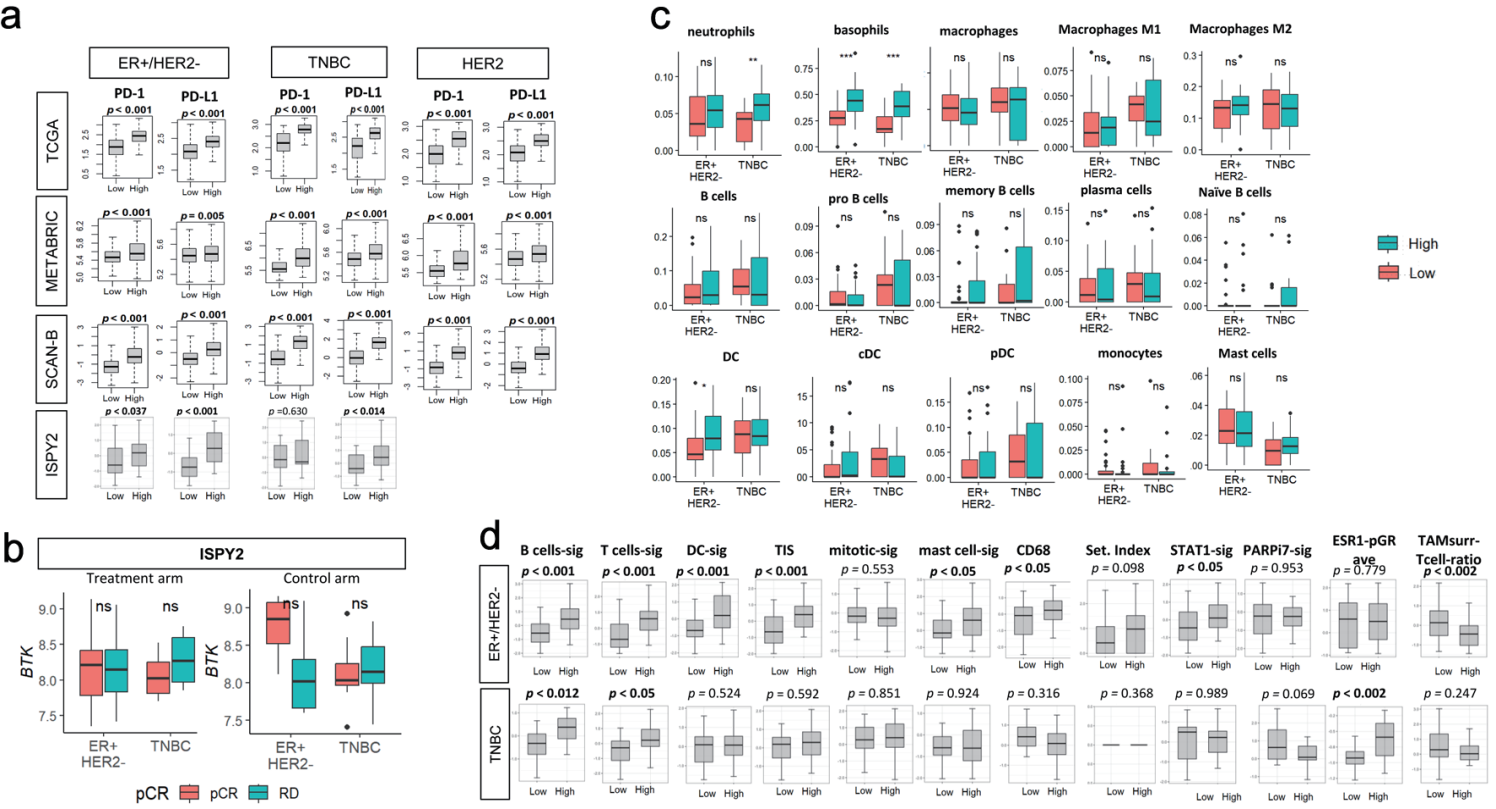
Association of pre-treatment tumor BTK expression with immune checkpoint molecule gene expressions, with response to immunotherapy or chemotherapy, with immune cell infiltrations and with immune-related gene signatures in the I-SPY2 cohort. (a) PD-1 or PD-L1 expressions by low and high BTK expressions, using median value as cut-off, for TCGA, METABRIC, SCAN-B, and I-SPY2 cohorts by subtypes. (b) Tukey boxplots of BTK expression by pathological complete response (pCR) or residual disease (RD) after treatment (durvalumab/olaparib and chemotherapy, n = 71, left panel) or control (chemotherapy alone, n = 34, right panel) arms by subtypes in the I-SPY2 trial. The “ns” (not significant) Mann-Whitney U test P value > 0.05. (c) Tukey boxplots display the relative expression of various immune cell populations in pretreatment tumor biopsies from patients in the I-SPY2 trial by subtypes (ER^+^/HER2^-^ and TNBC). (d) Tukey boxplots display the relative expression levels of 12 immune-related gene signatures in tumors from I-SPY2 trial. The “ns” (not significant) and asterisks above the plots indicate the statistical significance: *P < 0.05, **P < 0.01, ***P < 0.001.

Given the strong association between BTK expression and immune activation, we further interrogated its relationship with immune checkpoint molecules in TCGA, METABRIC, and SCAN-B, as well as with mast cell, myeloid, and B-cell signatures and immune-related metrics previously reported in the original I-SPY2 study. Across all three cohorts, and I-SPY2 cohort, and consistently across all subtypes, BTK-high tumors showed higher expression of the immune checkpoint molecules programmed cell death protein 1 (PD-1) and programmed cell death ligand 1 (PD-L1) (all P < 0.05, with only exception for PD1 expression in ER^+^/HER2^–^ in I-SPY2) (Fig. 7a).

Based on these findings, BTK expression was assessed by the response (pCR (n = 41) vs. residual disease (RD, n = 64)) to treatment arm (standard NAC plus durvalumab (PD-L1 inhibitor) and olaparib (Poly(ADP-ribose) polymerase (PARP) inhibitor)) or control arm (standard NAC) by subtypes in I-SPY2 trial (GSE173839 cohort). BTK mRNA expression did not significantly differ between pCR and RD groups in neither ER^+^/HER2^–^ nor TNBC subtypes in both arms, although BTK levels were remarkably lower in RD after standard NAC in ER^+^/HER2^–^ compared to pCR (Fig. 7b). Comparable results were observed in the control cohort, indicating no association between BTK expression and pCR with PD-L1–directed therapy. Therefore, BTK levels were not associated with response to I-SPY2 regimen.

Since BTK expression was associated with immune activity and infiltration of the immune cells, it was of interest to investigate the relationship between BTK expression and immune cell infiltrations in I-SPY2 cohort (Fig. 7c). High expression of BTK was associated with neutrophils in TNBC (P < 0.01), basophils in both ER^+^/HER2^–^ and TNBC (P < 0.001), and total DCs in ER^+^/HER2^–^ (P < 0.01). Lastly, we investigated the relationship between BTK expression and immune-related metrics previously reported in the original I-SPY2 study (Fig. 7d). In ER^+^/HER2^–^ tumors, high expression of BTK was significantly correlated with B-cell signature, T-cell signature, DC signature, tumor infiltration signature (TIS), mast cell signature, CD68, STAT1 signature, and TAMsurr/T cell ratio (all P < 0.05). In TNBC, only B-cell signature, T-cell signature, and ESR1-pGR-avg significantly correlated with BTK high expression (all P < 0.05).

## Discussion

In this multi-cohort analysis incorporating TCGA, METABRIC, SCAN-B, and multiple NAC and immunotherapy datasets, we comprehensively investigated the clinical, molecular, and immunologic implications of BTK expression in BC. Our findings demonstrate that although BTK is not predictive of survival or response to chemotherapy or immunotherapy, its expression is strongly associated with tumor subtype, cellular proliferation, and immune activation, particularly immune cell infiltrations in the TME.

Despite its well-established role in hematological malignancies, BTK expression showed no significant association with AJCC stage, DFS, DSS, or OS across the three large BC cohorts. This pattern was consistent across molecular subtypes, suggesting that, unlike leukemia and lymphoma, BTK may not be a major driver of disease progression in BC. In contrast, Bi et al [47] analyzed 551 lung adenocarcinoma cases from TCGA cohort and reported that BTK expression was negatively correlated with the clinical pathologic characteristics (clinical stage, distant metastasis) but positively associated with improved survival of lung cancer patients. Furthermore, their analysis of TME revealed that immune-related signaling pathways, such as allograft rejection, complement, and interferon response, were significantly enriched in the BTK high expression group. These findings are consistent with our results. GSEA of BTK high breast tumors revealed robust enrichment of immune-related pathways, including allograft rejection, complement, IFN-α, IFN-γ, IL6/JAK/STAT3, inflammatory response, and TNF-α/NF-κB pathways. Deconvolution analyses demonstrated greater infiltration of macrophages, neutrophiles, DCs, CD8^+^, CD4^+^ T cells, and B cells. Single cell RNA sequencing data further supported these observations, showing that BTK expression originates predominantly from myeloid cells and B lymphocytes. Collectively, these results identify BTK as a marker of an active immune TME in BC.

Our findings agree with previous publications regarding the relationship between BTK and immune cells. Several reports have demonstrated that BTK was overexpressed in certain solid tumors including BC, as well as in peripheral immune components of the TME such as DCs, macrophages, MDSCs, and endothelial cells [48, 49]. These observations suggest that BTK expression in BC originates predominantly from TME, with only a minor contribution from epithelial tumor cells. Additionally, a truncated isoform, termed BTK-C, has been detected in BC tissue [50, 51].

It is now well established that the immune microenvironment plays a critical role in shaping tumor behavior and is associated with improved survival and better responses to chemotherapy across multiple tumor types including BC. TILs have been consistently linked to favorable outcomes in HER2-positive and TNBC subtypes in both the early-stage and advanced disease settings [52–54]. Large-scale prospective clinical trials such as the BIG 02-98 [55], the FinHER [56], and the ECOG [57] have repeatedly shown that increased TILs infiltration correlate with improved DFS and OS.

Multiple studies have demonstrated that TILs can serve as a predictor of therapeutic response. BCs with high TILs were associated with significantly better pCR rates following anthracycline and taxane combination NAC among 1,058 patients in the GeparDuo and GeparTrio trials [58]. In contrast, the predictive value of TILs for pCR in ER^+^HER2^–^ subtypes remains a subject of debate [17, 59]. Denkert and colleagues [60] conducted the largest pooled analysis to date, evaluating the predictive and prognostic relevance of TILs concentration using pretreatment H&E-stained slides of core biopsies, from 3,771 patients enrolled in six randomized neo-adjuvant BC trials. Their study confirmed that higher TILs abundance strongly predicted pCR, independent of subtype. Furthermore, achieving pCR was associated with improved DFS in patients with TNBC and HER2-positive subtypes, but not in a small luminal–HER2^–^ subgroup. In the advanced TNBC tumors, data from phase 2 KEYNOTE-086 study (NCT02447003) of pembrolizumab monotherapy, similarly demonstrated that higher TIL concentrations were associated with improved responses [61].

In contrast to these reports, our results did not demonstrate a survival or therapeutic benefit despite the presence of highly enriched immune microenvironment. BTK expression, although strongly associated with immune activation, was not predictive of response to either NAC or immunotherapy. Across 10 NAC datasets, BTK expression showed no consistent association with pCR, except for marginal correlations observed in ER^+^ and HER2-positive tumors in the GSE2506 cohort.

MDSCs are a heterogeneous population of immature myeloid cells that have potent immune suppressive activity [62]. Recent studies have highlighted the critical role of MDSCs in tumor progression and suppression of antitumor immunity [63, 64]. BTK is known to be highly expressed by MDSCs in multiple murine tumor models as well as human MDSCs and plays a role in MDSC development and activation [65, 66]. In the current study, BTK expression closely correlated with MDSC infiltrations in BC regardless of subtypes, which aligns with previous studies. To ensure robustness and biological validity, we estimated the MDSCs infiltration using multiple independently derived gene expression signatures. Collectively, these data provide a mechanistic framework linking BTK signaling to immune escape in BC and its association with increased MDSCs abundance suggests a role in fostering an immunosuppressive TME that limits effective antitumor immunity.

Ibrutinib was able to inhibit the phosphorylation of BTK in both murine and human MDSCs [67]. Varikut et al [68] showed that ibrutinib suppresses BC growth and metastasis by reprogramming MDSCs into mature DCs, thereby enhancing Th1 and cytotoxic T-lymphocyte responses. Related to this, MDSCs are recognized as a significant barrier to immune checkpoint inhibitor (ICI) efficacy [69]. Indeed, several studies have now confirmed that BTK can regulate MDSCs by using the BTK inhibitor ibrutinib which was found to block MDSC function and reversed MDCS-medicated inhibition of T cells and NK cells [67, 70]. Ibrutinib inhibited *in vitro* generation of human MDSC, and pretreatment of MDSC with ibrutinib significantly impaired nitric oxide production and migration. Ibrutinib treatment of tumor-bearing mice reduced splenic and tumor MDSC frequencies and enhanced the efficacy of anti-PD-L1 therapy [71, 72]. Our clinical analysis mirrors this nuance. Although BTK high tumors demonstrate broad immune enrichment, increased myeloid cell infiltrations including MDSCs, and immune checkpoint molecule expressions, BTK expressions were no different by the response to ICIs in I-SPY2 trial. This apparent paradox supports the interpretation that BTK marks tumors with heightened immune infiltration accompanied by concurrent immunosuppressive mechanism, driven in part by MDSCs and other suppressive myeloid populations. Importantly, the coexistence of elevated PD-1/PD-L1, cytolytic activity and myeloid enrichment suggests that BTK expression reflects immune activation constrained by dominant myeloid-mediated suppression rather than effective antitumor immunity. This provides a biologically plausible explanation for the lack of direct association with immunotherapy response and supports a therapeutic rationale for combining BTK inhibitors with immune checkpoint blockade to relieve myeloid-driven T-cell suppression and potentially enhance immunotherapy efficacy in BC.

There are several limitations in this study. First, our analyses relied on retrospective datasets with heterogeneity in treatments regimens and clinical data and are subject to selection bias. Although we included multiple large patient cohorts, they differed considerably in patient demographics, clinical characteristics, and sample sizes. Additional confounders such as tissue handling, sample quality, tumor purity, normalization methods, and variability in the portion of tumor sampled may also have influenced our results. These factors could underrepresent tumor heterogenicity and the full spectrum of BTK-related biology. Future investigations incorporating single cell RNA sequencing or spatial transcriptomic approaches will be needed to provide a more detailed view of the TME and BTK function. Subtype-specific analysis would yield further insights but were limited by the available sample sizes. Similarly, the use of median cutoff to define BTK-high versus BTK-low tumors may not represent the most clinically relevant threshold, and alternative cutoffs require validation. Second, while BTK expression was measured at transcriptomic level, protein level validation in large BC cohorts is needed. Third, the functional role of BTK in distinct immune cell subsets within the breast TME remains to be clarified. Fourth, although the current study incorporated single cell data sets, most of our data were dominated by bulk analysis and insights into cell-specific BTK functions. Finally, the absence of *in vitro* experiments precludes direct mechanistic conclusions. Future studies integrating multiplex immunohistochemistry, spatial transcriptomics, and functional assays will be critical to dissect whether BTK activity is predominately pro-tumorigenic, immunosuppressive, or immunostimulatory in solid tumors. Despite these limitations, our analysis is strengthened by its focus on resected human tumor specimens, which provide data closely reflecting the *in situ* biology of BC.

In conclusion, BTK expression in BC is associated with aggressive histologic features and an immune active microenvironment with immune cell infiltrations but does not predict survival or response to NAC or immunotherapy. These findings suggest that BTK is best considered as an indicator of immune activation rather than as a prognostic indicator. However, given its immunomodulatory functions, BTK warrants further investigation as a potential therapeutic target, particularly in combination with immune checkpoint or other immune-directed therapies.

## Supplementary Material

Suppl 1 Estimated survival (Kaplan-Meier) with log-rank test P value and hazard ratio (HR) of disease-free survival (DFS), disease-specific survival (DSS), and overall survival (OS) for breast cancer subtypes groups of patients with low and high BTK expressions within the cohorts, using median value as cut-off.

Suppl 2 Association of tumor BTK expression with myeloid-derived suppressor cell (MDSC) gene expression signatures.

## Data Availability

The datasets analyzed in the current study are publicly available and were obtained from cBioportal, GEO, SCP, and Thorsson et al [37].
