# Supplementary material for: Expression of Bruton’s Tyrosine Kinase Reflects Immune Cells Infiltration and Cell Proliferation in Breast Cancer
Source: World J Oncol. 2026 Mar 5;17(2):209–22. doi: 10.14740/wjon2708 (PMC12978404; doi:10.14740/wjon2708)
Supplement: Suppl 1 — Estimated survival (Kaplan-Meier) with log-rank test P value and hazard ratio (HR) of disease-free survival (DFS), disease-specific survival (DSS), and overall survival (OS) for breast cancer subtypes groups of patients with low and high BTK expressions within the cohorts, using median value as cut-off. [file wjon-17-02-209-s001.pptx]

## Slide 1
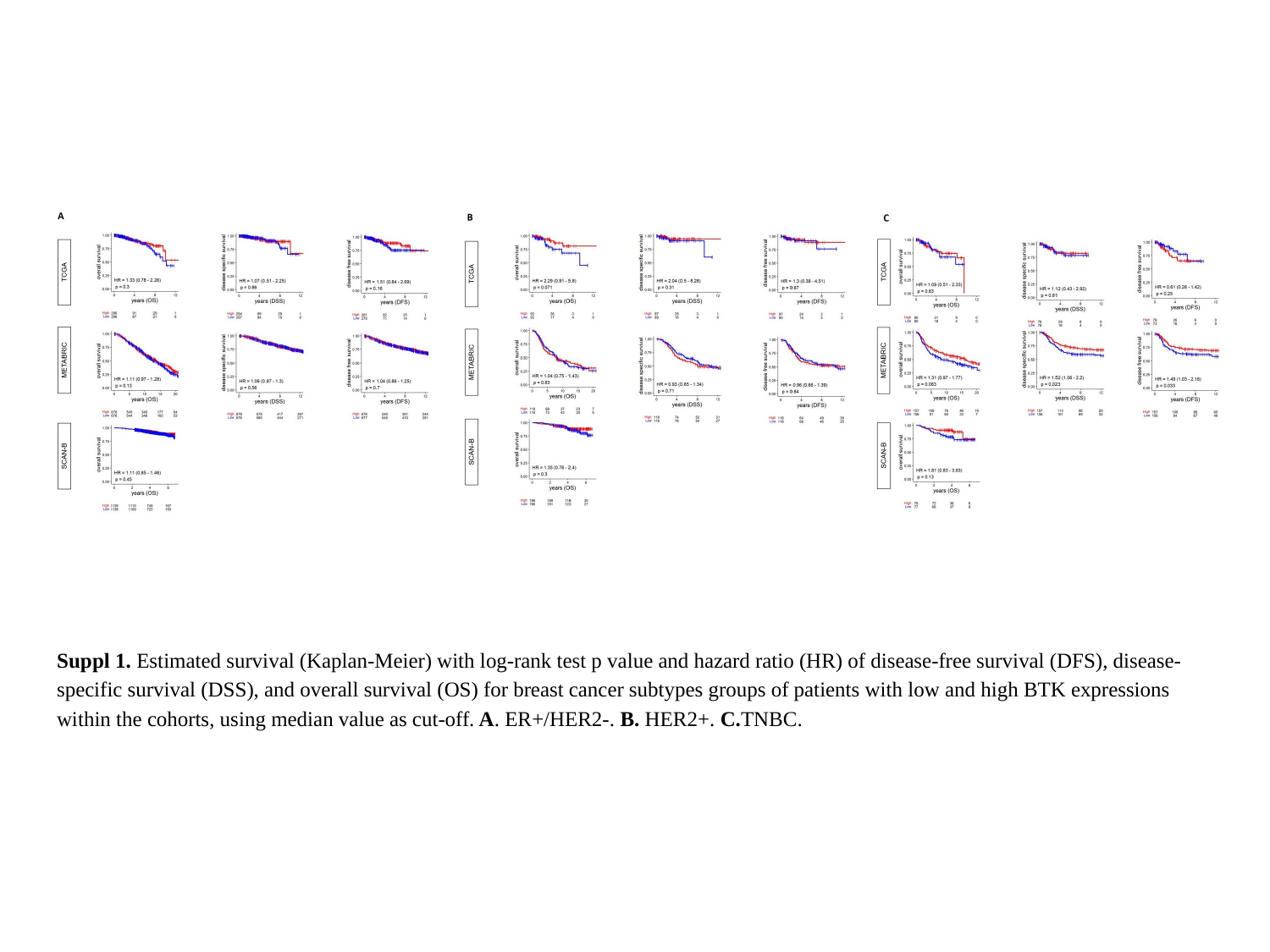

Suppl 1. Estimated survival (Kaplan-Meier) with log-rank test p value and hazard ratio (HR) of disease-free survival (DFS), disease-specific survival (DSS), and overall survival (OS) for breast cancer subtypes groups of patients with low and high BTK expressions within the cohorts, using median value as cut-off. A. ER+/HER2-. B. HER2+. C.TNBC.
