# Supplementary material for: Expression of Bruton’s Tyrosine Kinase Reflects Immune Cells Infiltration and Cell Proliferation in Breast Cancer
Source: World J Oncol. 2026 Mar 5;17(2):209–22. doi: 10.14740/wjon2708 (PMC12978404; doi:10.14740/wjon2708)
Supplement: Suppl 2 — Association of tumor BTK expression with myeloid-derived suppressor cell (MDSC) gene expression signatures. [file wjon-17-02-209-s002.pptx]

## Slide 1
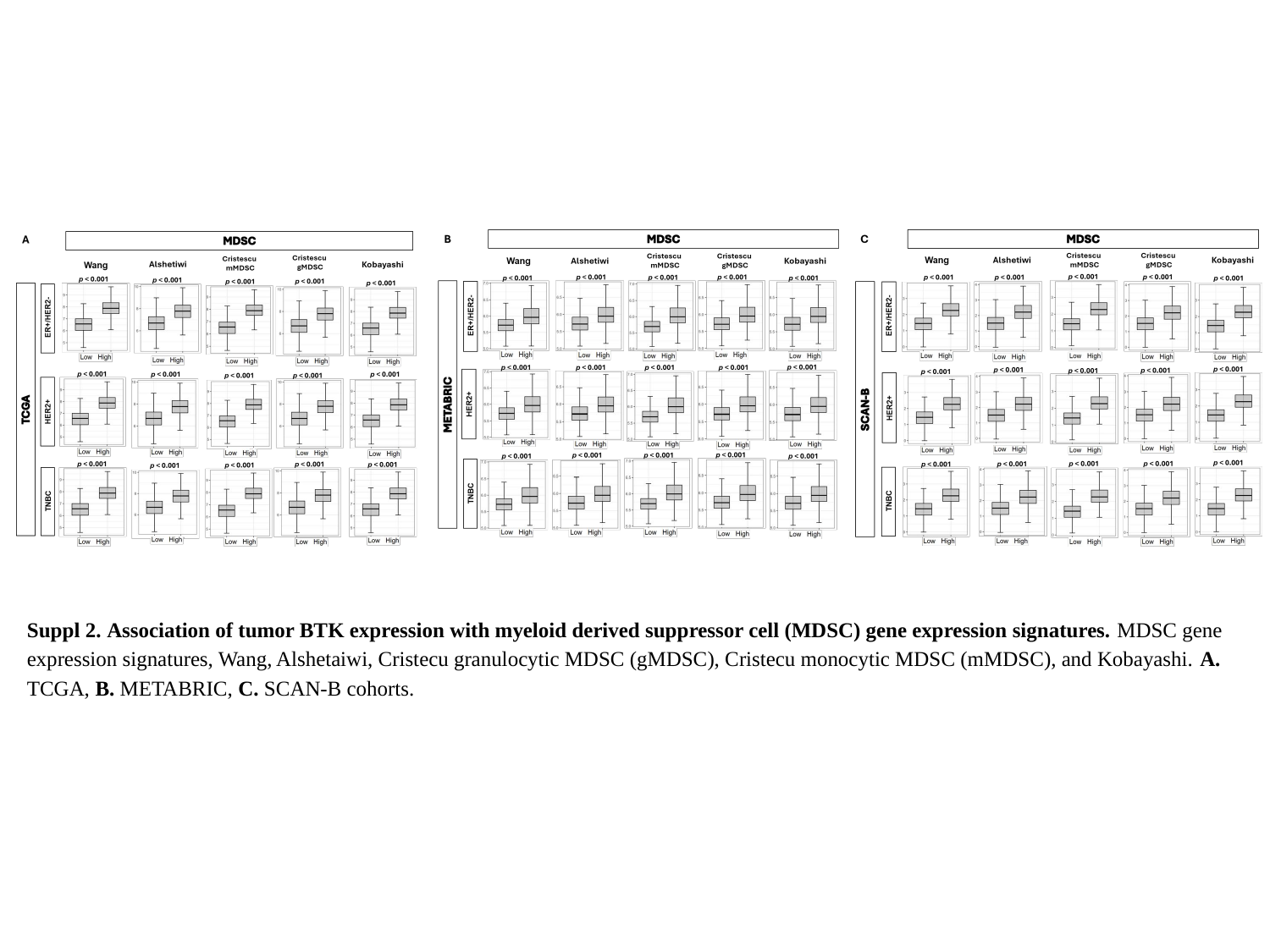

Suppl 2. Association of tumor BTK expression with myeloid derived suppressor cell (MDSC) gene expression signatures. MDSC gene expression signatures, Wang, Alshetaiwi, Cristecu granulocytic MDSC (gMDSC), Cristecu monocytic MDSC (mMDSC), and Kobayashi. A. TCGA, B. METABRIC, C. SCAN-B cohorts.
